# Clinical Challenges of Primary Diffuse Large B-Cell Lymphoma of the Dura: Case Report and Literature Review

**DOI:** 10.5402/2011/945212

**Published:** 2011-04-27

**Authors:** Rabih Said, Sanaa Rizk, Qun Dai

**Affiliations:** ^1^Department of Hematology and Oncology, Staten Island University Hospital, 256-C Mason Avenue, Staten Island, NY 10305, USA; ^2^Department of Internal Medicine, Staten Island University Hospital, NY 10305, USA

## Abstract

Primary dural lymphoma is a rare disease with more indolent clinical behavior compared to primary central nervous system lymphoma. The majority of the reported cases were indolent marginal zone lymphoma subtype with more predilections to the spine. Herein, we are presenting a case of intracranial, diffuse large B-cell lymphoma of the dura that was diagnosed and treated at our institution. We are presenting the challenges in the treatment based on a review of the literature.

## 1. Introduction

Primary leptomeningeal lymphoma (PLML) is a known, exceedingly rare subtype of Primary Central Nervous System Lymphoma (PCNSL), and it represents less than 0.1% of all Non-Hodgkin's Lymphomas (NHL) [1]. PLML is primarily a disease that originates from the meninges without any brain or systemic extension and/or involvement. It usually presents with nonspecific neurologic symptoms and signs such as headache, meningeal signs, and cranial nerve involvement with poor prognosis [2]. 

Primary Dural Lymphoma (PDL), a reported subentity of PLML, is described as dura matter involvement with no systemic disease. PDL is extremely rare disease, and it represents less than 1% of all brain lymphomas [7–9]. 

The reported cases of PDL describe predominantly a low-grade marginal zone lymphoma (MZL) with handful of reported cases of other histologic subtype of lymphoma [6, 10]. Herein, we reported a case of primary epidural diffuse large B-cell lymphoma of a young lady diagnosed and treated at Staten Island University Hospital.

## 2. Case Presentation

A 42-year-old Hispanic lady with no previous medical or surgical history presented to our hospital in April 2008 complaining of right upper and lower extremity weakness and numbness of 2-week duration associated with moderate headaches and retro-orbital pain. 

**Figure 1:**
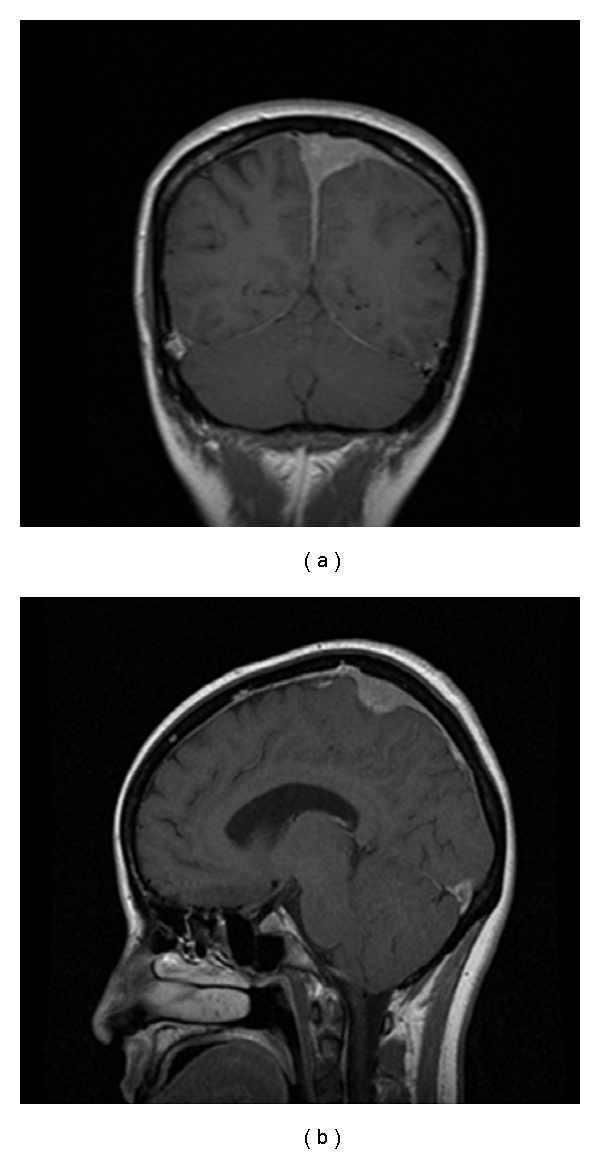
MRI at diagnosis showing broad-based attachment lesion to the meninges with indentation upon the adjacent brain parenchyma.

 Her physical exam was not significant except for 3/5 motoricity in RUE and RLE. The rest of neurologic exam (sensation, cranial nerves, DTR, and cerebellar) was normal. Complete blood count showed WBC 8600/*μ*L, granulocytes 65.6%, lymphocytes 25.9%, monocytes 7.8%, basophiles 0.5% and eosinophils 0.2%, hemoglobin 12.9 g/dL, hematocrit 36.9%, MCV 89 fl, and platelets 347/*μ*L. LDH level was 239 U/L (normal values 60–200 U/L). A CT brain with no contrast showed hyperdense extraaxial mass of the left vertex abutting the superior sagittal sinus (2.5 × 2.2 × 1.5 cm) likely reflecting meningioma. An MRI with and without gadolinium showed 2.4 cm extraaxial mass at the vertex compatible with meningioma which showed intermediate signal on T1 and T2 sequences and enhanced homogeneously, the sagittal sinus was compressed by the mass, and the orbital and soft tissue of the skull base was normal (Figure 1). She was seen by neurosurgery team. A left parietal craniotomy with mass resection was done 3 days later. During the surgery, it was obvious that the dura was thickened and perhaps involved by the tumor. This was carefully taken off the brain and parenchyma. At midline, the main portion of the tumor was identified. 

The pathology revealed malignant lymphoma, large cell type, diffuse large B-cell immunophenotype with no brain parenchyma identified indicating a dural diffuse large B-cell lymphoma. Immunochemical studies showed positive CD20, CD45, CD3, CD5, CD15, BCL-6 and negative CD30, EMA, BCL-2. In situ hybridization showed kappa-positive clonal population for this large lymphoma. 

A lumbar puncture post craniotomy was subsequently performed. Gram stain, culture, cytology and flow-cytometry of the CSF were all normal. Spinal MRI was negative.

Staging work up done, including a normal bone marrow aspirate and biopsy, normal routine cytogenetic and normal CT scan of the chest, abdomen and pelvis as well as whole body PET SCAN. Ophthalmologic exam was normal. At this point, the patient diagnosis was stages IE diffuse large B-cell Non-Hodgkin's lymphoma involving the dural-based on Ann Arbor staging system.

The patient was started adjuvant chemotherapy consisting of high-dose methotrexate-based regimen every 2 weeks for four cycles (MTX 3 gram/m² on day 1, vincristine 1.4 mg/m² on day 1 only cycles 1 and 3, procarbazine 100 mg/m² day 1 through day 7 on cycles 1 and 3 only) in May 2008. 

After four cycles, a repeated MRI brain with and without contrast revealed patchy meningeal enhancement sub-adjacent to the craniotomy site. A repeat PET SCAN revealed hypermetabolic uptake in the previous left high parietal craniotomy site, left posterior frontal sulcus and left parietal sulci with max SUV value of 15,6. FDG uptake was higher on this current study indicating residual disease. Therefore four cycles of R-CHOP protocol every 3 weeks were started in July 2008 (rituximab 375 mg/m² intravenous on day 1, cyclophosphamide 750 mg/m² intravenous on day 1, adriamycin 50 mg/m² intravenous on day 1, vincristine 1.4 mg/m² intravenous on day 1, and prednisone 100 mg oral day 1 through day 5).

**Figure 2:**
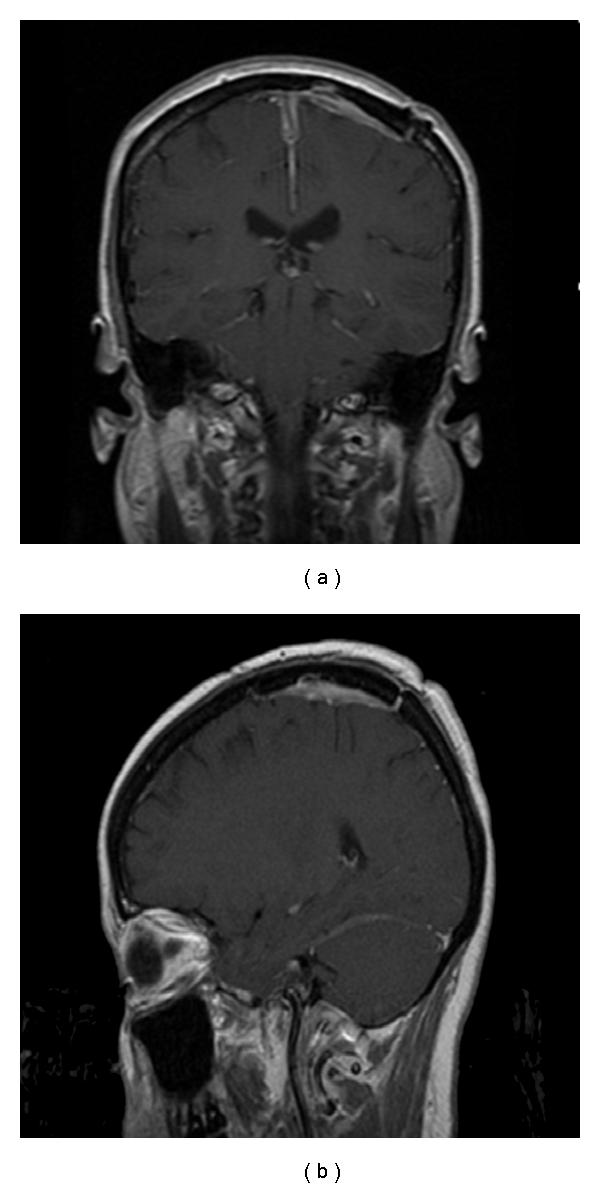
MRI posttreatment showing resolution of the disease with postsurgical changes.

 A brain MRI was repeated afterward showing focal areas of low T1, and high T2 high flair intensity with no enhancement was seen in left high medial frontal parietal parenchyma mostly representing postsurgical changes (Figure 2). There was no evidence of recurrent or residual disease. No radiation therapy was given at this time. 

After the last cycle of R-CHOP (October, 2008), she has been followed every 3 months that subsequently spaced out to currently every 6 months with repeated blood work and brain MRI that remained unchanged for 34 months. She is been active with no neurological deficit and no systemic dissemination of the disease during all this period of time until this report was concluded.

## 3. Discussion

PLML was first described, in the early 1990s, histologically as indolent type of B-cell lymphoma mainly extranodal marginal zone predominantly seen in middle-aged woman [2, 6, 10]. The main characteristic of PLML is the absence of systemic as well as central nervous system involvement. 

Since lymphoid tissue is absent in the dura, the pathogenesis of PDL is still unclear, many hypotheses have been formulated including the role of chronic inflammatory process, chronic infection, autoimmune disease and the meningoepithelial component [11–18]. 

Few case reports with other histological subtypes have been described including, follicular, Hodgkin, and diffuse large B-cell [3–6, 19]. 

Our case reported herein, is diagnosed as DLBCL of the dura matter based on the histologic and immunohistochemistry features of the malignant cells found only in the dura matter as well as the absence of systemic and CNS parenchymal involvement. 

Table 1 summarizes the reported cases of DLBCL of the dura matter. Compared to previous reported dural DLBCL, our case is the 1st report of intracranial location. 

 Meningioma is the first differential diagnosis with major similarities in radiographic and clinical presentations. The symptoms are usually dependant on the location of the tumor, including headaches, seizures, focal sensory or motor deficits, and visual disturbances, as well as nausea, vomiting, and ataxia [20, 21]. Cranial nerves dysfunction causing bilateral visual and hearing loss due to progressive dural involvement have been reported. Radicular pain and paraparesis are the most presenting symptoms in the case of spinal PDL [6]. 

Single or multiple dural-based extraaxial lesions that diffusely enhance after gadolinium injection are the typical radiologic feature on MRI. The presence of vasogenic edema and parenchymal brain invasion with a fuzzy tumor-brain interface is in favor of PDL more than meningiomas [20]. The involvement of leptomeningeal space makes the evaluation of CSF critical [20]. 

The clinical challenges that we face in managing our patient can be summarized in 3 categories: (1) the type of systemic adjuvant chemotherapy, (2) the role of radiation therapy and (3) the role of prophylactic intrathecal chemotherapy.

Due to the paucity of cases described in the literature, there is no standard treatment available for PLML. Although surgical treatment of dural MZL is considered the standard modality of treatment, complete resection can be technically difficult. Complete surgical resection was achieved in our patient, and this could be a contribution to the good overall response.

The first challenge was the type of systemic adjuvant chemotherapy and whether we should treat the patient as a parenchymal CNS lymphoma with high-dose methotrexate regimen or with R-CHOP regimen given the fact that this is an extraparenchymal disease. The patient was given high dose methotrexate regimen because of its good central nervous system penetration. We thought that this characteristic of methotrexate could be a helpful factor in avoiding radiotherapy. In addition, the patient had an isolated dural lesion with no other systemic disease which also made methotrexate a more reasonable treatment; however, there is no standard guideline in term of regimen the decision should be made individually. The patient later received R-CHOP because MRI finding suggested residual disease. Currently, there is no gold standard imaging study which can precisely differentiate between residual disease and post surgical inflammatory changes, and when it is not clear, waiting and repeat image is often the approach. However, we did not feel that waiting would be an appropriate strategy for our patient. On the other hand, pursuing another brain biopsy was not feasible either. Therefore, we decided to give her second round of chemotherapy with R-CHOP, which can further eradicate the residual disease.

Regarding the imaging modality to follow CNS lymphoma, we feel that enhanced MRI of the brain is more reliable. Data for use of PET scan in primary CNS lymphoma are lacking; PET can assess the metabolism within tumor and normal tissue by using radio-labeled tracers. However, false positivity is of concern especially in the brain. The brain directed PET may improve reading. 

The other question is the role of radiation therapy in the management of our patient. Cranial radiation therapy has also been used for patients with primary CNS lymphoma [22] after high dose systemic methotrexate chemotherapy. Three to 4 cycle R-CHOP followed by involved field radiation therapy has produced excellent outcome in patients with limited stage systemic DLBCL [23]. Should radiation be given to our patient? There is no definitive answer; we felt that benefit and long term side effect from brain radiation therapy should be carefully weighed in this young otherwise healthy patient. Given she has received high dose methotrexate which gives good blood-brain barrier penetration and given she has isolated low volume disease; we thought that she could avoid radiation up front. Radiation can be deferred as salvage treatment if she develops relapse disease. 

The role of intrathecal chemotherapy in the management of PLML is unclear. We felt that our patient will not benefit much from intra-thecal methotrexate because of the negative CSF analysis for malignant cell, the use of high dose systemic methotrexate regimen which has good blood-brain barrier penetration and the known inefficacy of the prophylactic intrathecal methotrexate in systemic DLBCL [24].

## 4. Conclusion

Although it is a rare entity, primary dura matter lymphoma exists. No standard of care is optimal due to its paucity; therefore, any case is encouraged to be reported. From our experience, the management should be a multidisciplinary approach that involves the neurosurgery, radiation, and medical oncology teams. Until more data are acquired, the optimal treatment should be individualized.

## Figures and Tables

**Table 1:** Reported cases of primary dural DLBCL.

Reference	Age (years)	Sex	Location	Therapy	Survival status (months)	Residual disease
Epelbaum (1986) [3]	73 53 45	F M M	Spine Spine Spine	S + R S + R + C S + R + C	D (26) D (20) A (42)	− + −
Eeles (1991) [4]	71 61 54	M F M	Spine Spine Spine	S + C S + R + C S + R + C	D (2) D (44) A (43)	+ + −
Lyons (1992) [5]	68 73 75 80	F M M M	Spine Spine Spine Spine	S + R S + R S + R S + R	A (71) D (24) D (12) D (10)	− + − −
Miranda (1996) [6]	36 46	F F	Spine Spine	S + R S + R + C	A (39) A (13)	

M: male; F: female; S: surgery; R: radiation therapy; C: chemotherapy; D: dead; A: alive.

## References

[1] Maher EA, Fine HA (1999). Primary CNS lymphoma. *Seminars in Oncology*.

[2] Lachance DH, O’Neill BP, Macdonald DR (1991). Primary leptomeningeal lymphoma: report of 9 cases, diagnosis with immunocytochemical analysis, and review of the literature. *Neurology*.

[3] Epelbaum R, Haim N, Ben-Shahar M, Ben-Arie Y, Feinsod M, Cohen Y (1986). Non-Hodgkin’s lymphoma presenting with spinal epidural involvement. *Cancer*.

[4] Eeles RA, O’Brien P, Horwich A, Brada M (1991). Non-Hodgkin’s lymphoma presenting with extradural spinal cord compression: functional outcome and survival. *British Journal of Cancer*.

[5] Lyons MK, O’Neill BP, Marsh WR, Kurtin PJ (1992). Primary spinal epidural non-Hodgkin’s lymphoma: report of eight patients and review of the literature. *Neurosurgery*.

[6] Miranda RN, Glantz LK, Myint MA (1996). Stage IE non-Hodgkin’s lymphoma involving the dura: a clinicopathologic study of five cases. *Archives of Pathology and Laboratory Medicine*.

[7] Jellinger K, Radaskiewicz TH, Slowik F (1975). Primary malignant lymphomas of the central nervous system in man. *Acta Neuropathologica. Supplementum*.

[8] Woodman R, Shin K, Pineo G (1985). Primary non-Hodgkin’s lymphoma of the brain. A review. *Medicine*.

[9] Zimmerman HM (1975). Malignant lymphomas of the nervous system. *Acta Neuropathologica. Supplementum*.

[10] Narberhaus B, Buxó J, Pérez De Olaguer J (1996). Primary dural lymphoma. *Neurologia*.

[11] Iwamoto FM, Abrey LE (2006). Primary dural lymphomas: a review. *Neurosurgical focus*.

[12] Kumar S, Kumar D, Kaldjian EP, Bauserman S, Raffeld M, Jaffe ES (1997). Primary low-grade B-cell lymphoma of the dura: a mucosa associated lymphoid tissue-type lymphoma. *American Journal of Surgical Pathology*.

[13] Isaacson PG, Du MQ (2004). MALT lymphoma: from morphology to molecules. *Nature Reviews Cancer*.

[14] Zucca E, Roggero E, Pileri S (1998). B-cell lymphoma of MALT type: a review with special emphasis on diagnostic and management problems of low-grade gastric tumours. *British Journal of Haematology*.

[15] Estevez M, Chu C, Pless M (2002). Small B-cell lymphoma presenting as diffuse dural thickening with cranial neuropathies. *Journal of Neuro-Oncology*.

[16] Miller M, Loffe V, Ruffin WK, Giri PG (2004). Primary MALT lymphoma of the dura in a patient with active scleroderma. *Clinical Advances in Hematology and Oncology*.

[17] Sanjeevi A, Krishnan J, Bailey PR, Catlett J (2001). Extranodal marginal zone B-cell lymphoma of malt type involving the cavernous sinus. *Leukemia and Lymphoma*.

[18] Kambham N, Chang Y, Matsushima AY (1998). Primary low-grade B-cell lymphoma of mucosa-associated lymphoid tissue (MALT) arising in dura. *Clinical Neuropathology*.

[19] Johnson MD, Kinney MC, Scheithauer BW (2000). Primary intracerebra Hodgkin’s disease mimicking meningioma: case report. *Neurosurgery*.

[20] Iwamoto FM, DeAngelis LM, Abrey LE (2006). Primary dural lymphomas: a clinicopathologic study of treatment and outcome in eight patients. *Neurology*.

[21] Tu PH, Giannini C, Judkins AR (2005). Clinicopathologic and genetic profile of intracranial marginal zone lymphoma: a primary low-grade CNS lymphoma that mimics meningioma. *Journal of Clinical Oncology*.

[22] DeAngelis LM, Seiferheld W, Clifford Schold S, Fisher B, Schultz CJ (2002). Combination chemotherapy and radiotherapy for primary central nervous system lymphoma: radiation Therapy Oncology Group study 93-10. *Journal of Clinical Oncology*.

[23] Horning SJ, Weller E, Kim K (2004). Chemotherapy with or without radiotherapy in limited-stage diffuse aggressive non-Hodgkin’s lymphoma: Eastern Cooperative Oncology Group Study 1484. *Journal of Clinical Oncology*.

[24] Boehme V, Schmitz N, Zeynalova S, Loeffler M, Pfreundschuh M (2009). CNS events in elderly patients with aggressive lymphoma treated with modern chemotherapy (CHOP-14) with or without rituxima: an analysis of patients treated in the RICOVER-60 trial of the German High-Grade Non-Hodgkin Lymphoma Study Group (DSHNHL). *Journal of the American Society of Hematology*.

